# Effects of Topical Gabapentin on Ocular Pain and Tear Secretion

**DOI:** 10.3389/fphar.2021.671238

**Published:** 2021-06-07

**Authors:** Maurizio Cammalleri, Rosario Amato, Melania Olivieri, Salvatore Pezzino, Paola Bagnoli, Massimo Dal Monte, Dario Rusciano

**Affiliations:** ^1^ Department of Biology, University of Pisa, Pisa, Italy; ^2^ Sooft Italia SpA, Research Center, Catania, Italy

**Keywords:** neuropathic ocular pain, dry eye syndrome, corneal sensitivity, lacrimal gland, autonomous nervous system, aquaporin 5, corneal epithelial cells, PKA/CREB pathway

## Abstract

Neuropathic ocular pain is a frequent occurrence in medium to severe dry eye disease (DED). Only palliative treatments, such as lubricants and anti-inflammatory drugs, are available to alleviate patients’ discomfort. Anesthetic drugs are not indicated, because they may interfere with the neural feedback between the cornea and the lacrimal gland, impairing tear production and lacrimation. Gabapentin (GBT) is a structural analog of gamma-amino butyric acid that has been used by systemic administration to provide pain relief in glaucomatous patients. We have already shown in a rabbit model system that its topic administration as eye drops has anti-inflammatory properties. We now present data on rabbits’ eyes showing that indeed GBT given topically as eye drops has analgesic but not anesthetic effects. Therefore, opposite to an anesthetic drug such as oxybuprocaine, GBT does not decrease lacrimation, but–unexpectedly–even stimulates it, apparently through the upregulation of acetylcholine and norepinephrine, and by induction of aquaporin 5 (AQP5) expression in the lacrimal gland. Moreover, data obtained *in vitro* on a primary human corneal epithelial cell line also show direct induction of AQP5 by GBT. This suggests that corneal cells might also contribute to the lacrimal stimulation promoted by GBT and participate with lacrimal glands in the restoration of the tear film, thus reducing friction on the ocular surface, which is a known trigger of ocular pain. In conclusion, GBT is endowed with analgesic, anti-inflammatory and secretagogue properties, all useful to treat neuropathic pain of the ocular surface, especially in case of DED.

## Introduction

Neural regulation plays an integral role in maintaining ocular surface homeostasis by tightly controlling lacrimal gland secretion of tear film containing water, electrolytes and a variety of proteins (Dartt, 2009). Disruption of ocular surface homeostasis as induced by altered activity of the feedback loop between the corneal surface and the lacrimal gland causes disturbing effects of which ocular pain is a major component. The cornea has the densest sensory innervation in the human body and has the potential to be a powerful producer of pain (Galor, 2019). In case of corneal injuries the ciliary nerves innervating the cornea increase their activity, finally resulting in corneal hypersensitivity (Joubert et al., 2019). Stimulation of corneal sensory nerves promotes lacrimal gland secretion by the so-called tear reflex. Sensory afferents from the nerve endings on the corneal surface stimulate efferent sympathetic and parasympathetic fibers of the facial nerve that innervate the lacrimal gland via feedback loops between the ocular surface, lacrimal gland and brain. Activated parasympathetic and sympathetic nerves release acetylcholine (Ach) and norepinephrine (NE), although other different peptides can also be involved in the autonomic transmission (Russo, 2017). Released neurotransmitters stimulate distinct receptors, thus triggering a cascade of second messenger components activating ion channels and pumps to cause electrolyte, water and protein secretion (Dartt, 2009). Significant involvement of water channels in regulating lacrimal fluid secretion has been demonstrated (Delporte, 2009) with a major role played by aquaporin 5 (AQP5) in both the cornea and the lacrimal gland (Verkman, 2003). A disturbance of the neural feedback loops between the ocular surface and lacrimal glands can contribute to corneal diseases such as dry eye disease (DED), in which both nociceptive and neuroptahic pain may be involved (Galor et al., 2015).

Generally speaking, DED is a multifactorial pathology affecting the ocular surface of the eye, associated with pain that may arise from inflammation, reduction in the volume and/or quality of tears, and damage to the sensitive nerve endings located in the cornea (Galor, 2019). The treatment of DED is palliative, using artificial tears that provide temporary symptomatic relief, but do not address the underlying pathophysiology of the syndrome. Ideally, a complete treatment for neuropathic pain in DED should address tear dysfunction and the associated inflammation, and provide an analgesic effect to soothe the pain without reducing lacrimation.

Different drugs are commonly used to treat ocular pain, including anti-inflammatory, anesthetic and analgesic drugs (Jacobs, 2017). Analgesic drugs include gabapentin (GBT), a structural analog of gamma-amino butyric acid that has been introduced as an adjunctive therapy in epilepsy and is presently widely used to treat several kinds of neuropathic pain. The possibility that GBT may counteract ocular pain has been suggested by several studies (Lichtinger et al., 2011; Pakravan et al., 2012; Wei et al., 2015; Ongun and Ongun, 2019; Yoon et al., 2020), since the first demonstration of systemic GBT efficacy in providing significant pain relief in glaucomatous patients (Kavalieratos and Dimou, 2008). At least some of the clinical effects of GBT are due to high affinity interactions with the α2δ1 auxiliary subunit of presynaptic voltage-gated calcium channels (Taylor and Harris, 2020). However, more work needs to be done to fully understand the mechanisms through which GBT may target ocular tissues, and ameliorate ocular pain. In this respect, the recent finding that eye drops containing GBT exert major anti-inflammatory activity *in vitro* and *in vivo* (Anfuso et al., 2017) suggests the possibility that the analgesic effect of GBT coupled to its anti-inflammatory properties may confer a better ability to an eye drop formulation to treat ocular pain.

The present study stems from the consideration that anesthetics are not best indicated to fight neuropathic ocular pain, especially in case of dry eye, because they are expected to blunt the nervous feedback between the cornea and the lacrimal apparatus, thus inhibiting lacrimation. GBT, on the other hand, being analgesic and not anesthetic, should be devoid of such negative effects on lacrimation. Therefore, we designed *in vivo* experiments in the rabbit, aimed at investigating whether GBT, topically administered, may blunt cornea hypersensitivity induced by formaldehyde and whether its analgesic efficacy interferes with tear secretion regulation. Our unexpected finding that GBT stimulates lacrimation prompted us to address the contribution of the autonomic innervation to GBT-associated regulation of tear production, together with the possibility that GBT might act by regulating AQP5 levels in the lacrimal gland. In these experiments, pretreatment with the anesthetic drug oxybuprocaine (benoxinate: BNX), a topical anesthetic that reduces basal lacrimation (Shiono, 1989) and is mainly used to blunt the activity of corneal nociceptors (Nakamachi et al., 2016), was used to evaluate whether GBT acts indirectly through a modulation of the tear reflex or directly onto the lacrimal gland. In addition, as corneal epithelial cells participate in determining the final tear composition by secreting proteins, electrolytes, and water (Santagati et al., 2005; Meng and Kurose, 2013), further *in vitro* studies with primary human corneal epithelial (HCE-F) cells were carried out to show that GBT - but also BNX - might also directly trigger AQP5 expression through a molecular pathway involving PKA/CREB (Wang and Zheng, 2011).

## Materials and Methods

### Animals

New Zealand albino rabbits (32 males and 37 females, body weight 2.54 ± 0,26 kg, aged 170–190 days) were purchased from a local supplier. Animals were kept at a temperature of 22°C and a relative humidity of 50%. Each rabbit was kept in a single cage and provided with standard rabbit feed and drinking water.

### Cultured Cells

Primary HCE-F cells (Cristaldi et al., 2020) were seeded in complete culture medium at a density of 5 × 10^5^ cells/well in a six-well plate and left to adhere overnight in the incubator at 37°C and 5% CO_2_. In a first set of experiments, cells were treated in serum-free medium (SFM) with GBT at 0.01, 0.1 or 1 mg/ml for 24 h. In rabbit corneal epithelial cells, GBT at 0.01 and 0.1 mg/ml did not affect cell viability, which was modestly affected by GBT at 1 mg/ml (Anfuso et al., 2017). In a second set of experiments HCE-F cells were treated in SFM with BNX (BP700; Sigma-Aldrich, St. Louis, MO, United States) at 0.03 or 0.15 mg/ml, either alone or in combination with 0.1 mg/ml of GBT for 12 h. Neither GBT nor BNX affected HCE-F cell survival at the tested concentrations. At the end of each incubation period cells were scraped and stored at -80°C until use.

### Pharmacokinetics

Thirty microliters of a GBT (sc-201481; Santa Cruz Biotechnology, Inc., Santa Cruz, CA, United States) formulation made 2% in phosphate buffered saline (PBS) were instilled twice at an interval of 2 min in each eye of six New Zealand white rabbits. At 30, 60, and 120 min, two animals per each time point were euthanized, and the four eyes dissected to take separately cornea, conjunctiva and aqueous humor. A fixed amount of acetonitrile and H_2_O (50:50) solution was added to the biological samples to extract GBT. After mechanical trituration by Ultra-turrax (cornea and conjunctiva) and subsequent sonication, samples were centrifuged at 10,000 rpm for 15 min, and GBT contained in the supernatant was quantified by HPLC/MS/MS using the Agilent 6410-A triple quadrupole instrument equipped with a Phenomenex Kinetex C18 column at 25°C, under isocratic conditions using 92% of Buffer A (5 mM ammonium formate pH = 3.0) and 8% of acetonitrile, at a flow rate of 0.1 ml/min. The system is equipped with a positive ionizing mode ESI interface, such that the mass transition for GBT is 172.2 → 154.2 m/z. The operational MS parameters of the instrument were: Gas Temperature 350°C; Gas Flow 8 L/min; Nebulizer 20 psi; Capillary 3500 V; Collision energy 30 V; Dwell-time 70 msec; Fragmentor 50 V. Calibration curves were made in blank tissue extracts (cornea, conjunctiva, aqueous humor) by adding exact amounts (between 2 and 400 ng/ml) of GBT. Values were expressed as ng/mg of tissue.

### Corneal Sensitivity

Corneas were sensitized according to a previously published protocol (Lai et al., 2013), with some modifications. Formaldehyde 0.5% (30 µl) was instilled in the right eyes and 1 min after application the eyes were washed with sterile saline to remove residual formaldehyde. Left eyes were left untreated as controls. After formaldehyde application, no evident signs of corneal or conjunctival toxicity including signs of chemical trauma, were observed. The corneal sensitivity was evaluated with a Cochet-Bonnet esthesiometer (nylon thread; 0.12 mm diameter; length variable between 5 and 60 mm; Luneau, Paris, France). The nylon filament of the esthesiometer touched the center of the cornea perpendicularly. A positive response was recorded if > 5 reflexes occurred in 10 consecutive touches. The longest filament length causing a positive result was considered the corneal sensitivity threshold. 5 min after formaldehyde instillation, 30 µl of sterile saline or 2% GBT were instilled in both eyes in the corneal-limbic junction. This route of administration was chosen because it is the intended way of human exposure to the test formulation. At progressing times corneal sensitivity was tested. Whether GBT might act as an anesthetic was evaluated by comparing the effects of 30 µl of either 2% GBT or 0.4% BNX instilled in the eyes of rabbits that did not receive formaldehyde. The number of stimuli necessary to induce a blinking reflex maintaining a fix length of 7.5 mm was counted over time. In any case, the number of mechanical stimuli applied was 10 in order to limit corneal damage.

### Schirmer’s Tear Test

The secretory effect of the test compounds was evaluated after the instillation of 30 µL of either 2% GBT or 0.4% BNX. In some experiments, BNX was applied 15 min before GBT instillation. Tear secretion was measured using commercially available Schirmer’s tear test strips (Contacare Ophthalmics and Diagnostics, Dunstable, United Kingdom), as previously reported (Honkanen et al., 2021). Briefly, in each eye the strip was placed in the mid portion of the lower lid at progressing times and tear production was recorded as the length of moistened strip at 1 min. Then, the heads of the strips were cut and stored at -80°C until use to evaluate tear protein concentration.

### Tear Protein Concentration

Tear proteins were extracted from the heads of the Schirmer’s tear test strips by elution with 100 µl of 100 mM ammonium bicarbonate containing 0.25% NP-40 with addition of proteinase inhibitors (Roche Applied Science, Indianapolis, IN), as previously described (Yu et al., 2018). Samples were incubated on a rotator overnight at 4°C. Then they were centrifuged, and protein concentration was measured with the Micro BCA Protein Assay (Thermo Fisher Scientific, Waltham, MA, United States).

### Tissue Harvesting

After the administration of GBT, BNX or BNX before GBT, at progressing times rabbits were narcotized with a mixture of ketamine (40 mg/ml) and xylazine (10 mg/ml) and sacrificed with an overdose of Nembutal (80 mg/kg). Untreated rabbits were sacrificed as well. Lacrimal glands, cornea and conjunctiva were removed without removal of the eye bulb. The brain was removed and hippocampus isolated. All tissues were rinsed in sterile saline and stored at −80°C until use.

### ELISA

Before the use, lacrimal glands were cut in smaller pieces, mixed, and randomly divided for ELISA or Western blotting. To determine the content of parasympathetic and sympathetic neurotransmitters, lacrimal glands were homogenized in PBS in the presence of proteinase inhibitors (Roche Applied Science). Homogenates were centrifuged at 5,000 rpm for 5 min and supernatants were immediately used. Protein concentration was measured with the Micro BCA Protein Assay (Thermo Fisher Scientific). Ach and NE content were evaluated by using commercially available ELISA kits, according to the manufacturer instructions (Acetylcholine ELISA kit, OKEH02568, Aviva Systems Biology, San Diego, CA, United States and NA/NE ELISA kit, MBS760375, MyBioSource, San Diego, CA, United States, respectively). Ach and NE levels were calculated by interpolating the absorbance of each sample against the respective calibration curves using lyophilized Ach and NE standards available in the kits.

### Quantitative Real Time PCR

To perform quantitative real time PCR (qPCR), total RNA was extracted and purified from rabbit tissues (hippocampus, lacrimal gland, cornea, conjunctiva) and HCE-F cells using the RNeasy Mini Kit (Qiagen, Valencia, CA, United States). First-strand cDNA was generated from 1 µg of total RNA (QuantiTect Reverse Transcription Kit, Qiagen). Real-time PCR amplification was performed with SsoAdvanced Universal SYBR Green Supermix (Bio-Rad Laboratories, Hercules, CA, United States) on a CFX Connect Real-Time PCR detection system and software CFX manager (Bio-Rad Laboratories). qPCR primer sets were chosen to hybridize to unique regions of the appropriate gene sequence: α2δ1 (Forward: 5′-AGA​CCC​TTC​ACT​GTT​GTG​GC-3′; Reverse: 5′-ACC​CAT​GGA​GAA​GCT​GGG​TA-3′); GAPDH (Forward: 5′-CCG​CTT​CTT​CTC​GTG​CAG​TG-3′; Reverse: 5′-CAA​TGC​GGC​CAA​ATC​CGT​T-3′). Samples were compared using the relative threshold cycle (Ct Method). The increase or decrease (fold change) was determined relative to the hippocampus after normalization to GAPDH, used as the housekeeping gene.

### Western Blotting

Samples (hippocampus, lacrimal glands, cornea, conjunctiva, or HCE-F cells) were homogenized in RIPA buffer containing phosphatase and proteinase inhibitor cocktails (Roche Applied Science) or in a nuclear and cytoplasmic extraction buffer (NE-PER Kit, 78,833, Thermo Fisher Scientific). Protein concentration was measured with the Micro BCA Protein Assay (Thermo Fisher Scientific). Samples (30 µg proteins each) were run on 4–20% or 4–12% SDS-PAGE gels and proteins were then transferred on nitrocellulose membranes. Blots were blocked for 1 h with 5% skim milk and incubated overnight at 4°C with the primary antibodies indicated in Table 1 using β-actin or histone H1 as loading controls. Blots were then incubated for 1 h with HRP-conjugated secondary antibodies (1:5,000) and developed with the Clarity Western enhanced chemiluminescence substrate (Bio-Rad Laboratories). Images were then acquired (ChemiDoc XRS+; Bio-Rad Laboratories). The optical density of the bands was evaluated (Image Lab 3.0 software: Bio-Rad Laboratories). Protein expression level was normalized against β-actin (non-phosphorylated targets) or total non-phosphorylated corresponding protein (phosphorylated targets).

**TABLE 1:** List of antibodies used in Western blot.

Antibody	Dilution	Source	Cat. No
Mouse monoclonal anti α2δ1	1:1,000	Thermo Fisher Scientific	MA3-921
Mouse monoclonal anti-AQP5	1:500	Santa Cruz Biotechnology, Inc	sc-514022
Rabbit polyclonal phosphor (Ser/Thr) PKA	1:1,000	Cell Signaling	9621S
Rabbit polyclonal anti-PKA C-alpha	1:1,000	Cell Signaling	4782S
Rabbit polyclonal anti-phospho CREB (Ser133) (87G3)	1:1,000	Cell Signaling	9198S
Mouse monoclonal anti-CREB (86B10)	1:1,000	Cell Signaling	9104S
Mouse monoclonal anti-β-actin	1:2,500	Sigma-Aldrich	A2228
Mouse monoclonal anti-Histone H1 clone AE-4	1:2,000	Sigma-Aldrich	05–457

### Statistics

Data were analyzed by the Shapiro-Wilk test to verify their normal distribution. Statistical signiflcance was evaluated with Prism 8.0.2 (GraphPad Software, Inc., San Diego, CA, United States) using one-way analysis of variance (ANOVA) followed by Tukey’s multiple comparison post-test or two-way ANOVA followed by Bonferroni multiple comparison post-test. Data are expressed as means ± SEM of the reported n values. Differences with *p* < 0.05 were considered significant. In compliance with the 3Rs principles for ethical use of animals in scientific research, an a priori power analysis was conducted using the software G*Power 3.0.10 (www.gpower.hhu.de) to determine the minimum number of animals necessary to obtain a statistical power of at least 0.80, with α = 0.05, in the presence of a large effect size as expected in these studies.

## Results

### Gabapentin Pharmacokinetics and Effects on Corneal Sensitivity and Lacrimation

**FIGURE 1:**
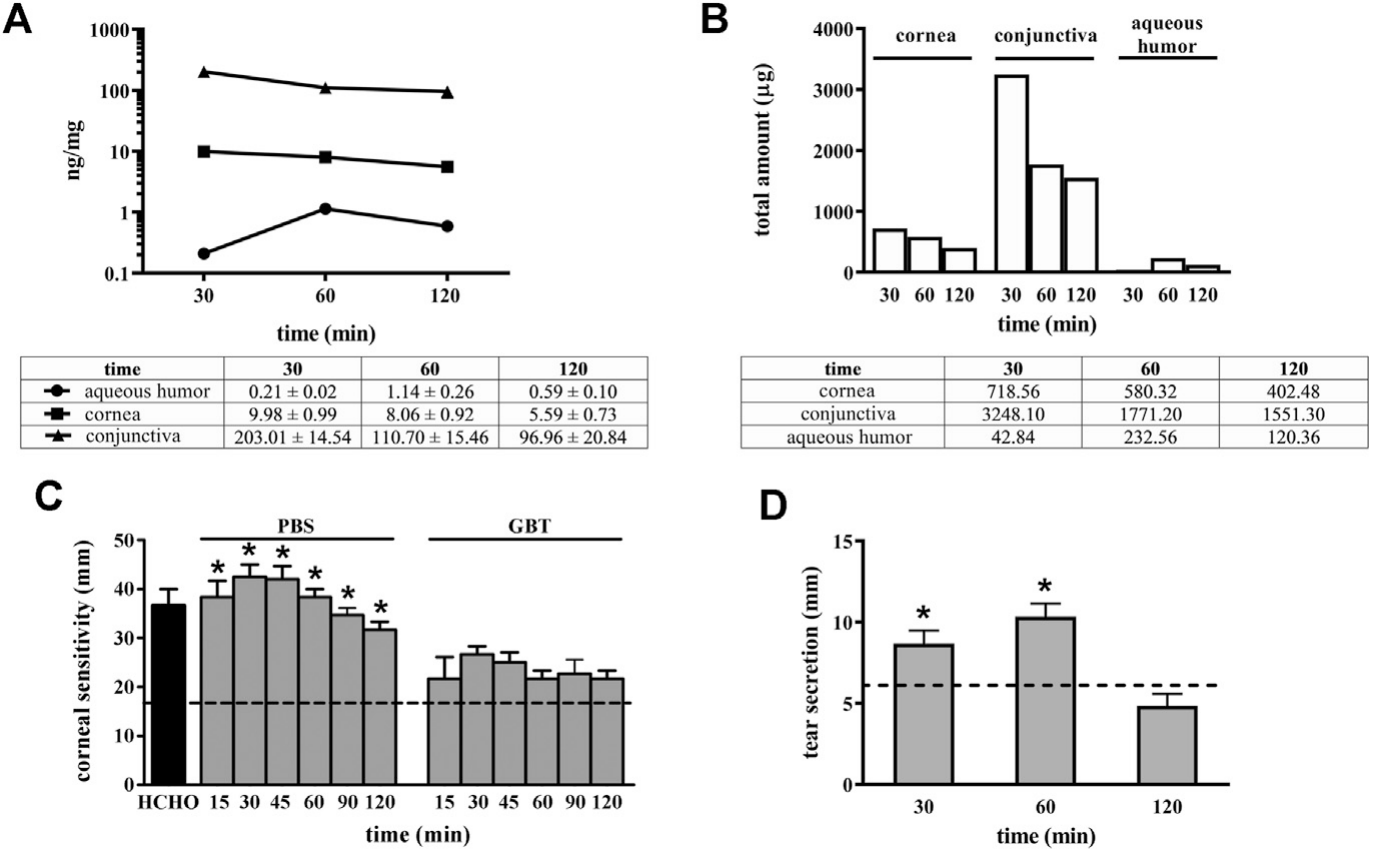
Pharmacokinetic profile of GBT (2% in PBS) in ocular tissues and GBT analgesic effects. GBT was measured in aqueous humor, cornea and conjunctiva at different times. For each tissue, concentration profiles derived from four samples per treatment are reported in ng/mg of tissue **(A)**, or as total estimated amount in each tissue **(B)**. **(C)** Effects of 2% GBT on corneal pain. Longitudinal evaluation of corneal sensitivity using the Cochet-Bonnet esthesiometry in rabbits treated with PBS or GBT after the instillation of 0.5% formaldehyde. The dotted line indicates the basal value. Differences between groups were tested for statistical significance using one-way ANOVA with Tukey’s multiple comparison post-test (*n* = 6). **(D)** Effects of 2% GBT in PBS on tear secretion. The dotted line indicates the basal value. Differences vs. basal secretion were tested for statistical significance using two-way ANOVA with Bonferroni multiple comparison post-test. *p < 0.05. All data are plotted as mean ± SEM.

The distribution of GBT eye drops (2% in PBS) has been evaluated in aqueous humor, cornea and conjunctiva. Figure 1A shows the GBT concentration per mg of tissue, which results always higher on the ocular surface (conjunctiva and cornea), though slowly decreasing with time (after 120 min there is, respectively, still 50 and 60% of the amount determined at 30 min), while the aqueous humor contains the lowest amount, peaking at 60 min, and then decreasing. Figure 1B illustrates the total amount calculated per tissue. To this purpose, we have experimentally estimated the average weight of each tissue in rabbits of 2.5 Kg and 3 months old at 72 mg for cornea, 160 mg for conjunctiva and 204 mg for aqueous humor, which is in line with the expectations (Struble et al., 2014).

It is thus evident that the most part of GBT (97.7% at 30 min, and 96.7% at 120 min) remains in the conjunctiva, from which it can be distributed to neighboring tissues, and the cornea contains most of the remaining amount (2.1% at 30 min, and 2.5% at 120 min).

GBT efficacy on neuropathic ocular pain is shown in Figure 1C. Normally, in untreated healthy rabbit eyes a thread length of 18 mm is the higher length able to induce a corneal reflex. After corneal injury with 0.5% formaldehyde, the corneal sensitivity increased by about 2-fold as compared to the basal level, and rabbit eyes responded with a blinking reflex at a thread length of about 40 mm. Control eye drops (PBS alone) did not affect this elevated corneal sensitivity that was instead significantly reduced by 2-fold (a response was elicited at a thread length of about 20 mm, similar to untreated control) after 2% GBT eye drops up to 120 min after their instillation.

In order to confirm that the analgesic effect of GBT eye drops did not decrease lacrimation, as it could be expected from a topical anesthetic, the effects on tear secretion of GBT eye drops was also evaluated. As shown in Figure 1D, 2% GBT not only did not decrease lacrimation, but–surprisingly–it significantly increased tear secretion in respect to the basal level at both 30 and 60 min, to lose its effect at 120 min.

**FIGURE 2:**
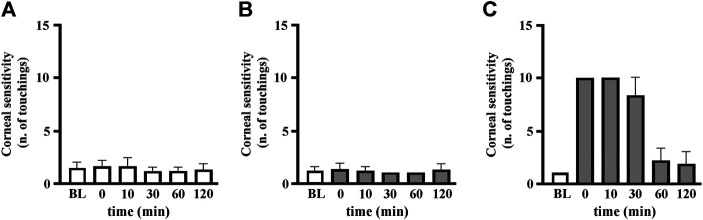
Analgesic and not anesthetic activity of topical GBT. Rabbit eyes (*n* = 6) were instilled with PBS **(A)**, 2% GBT **(B)** or 0.4% BNX **(C)**, and corneal sensitivity was evaluated at different times by the number of touchings at minimal extension (0.75 mm) eliciting a blinking reflex. BL, baseline.

Figure 2 shows how corneal sensitivity is modulated by GBT or by the administration of a classical ocular anesthetic (BNX) on naïve corneas. BNX is known to reduce basal lacrimation (Shiono, 1989) and is mainly used to blunt the activity of corneal nociceptors (Nakamachi et al., 2016). In this case, the thread of the esthesiometer was kept at a fixed short length of 7.5 mm, thus eliciting an immediate blinking reflex: one touch was already enough to stimulate a response (Figure 2A). The instillation of GBT did not change corneal sensitivity at this rude touch (Figure 2B), while the instillation of the anesthetic BNX made the cornea much less responsive to the stimulation with the short thread, requiring more than five touchings to elicit a blinking reflex (Figure 2C).

### Gabapentin Effect on Aqueous Tear Secretion and Protein Concentration

**FIGURE 3:**
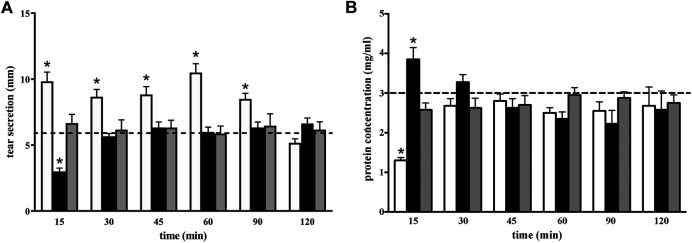
Longitudinal evaluation of aqueous tear secretion **(A)** and total protein concentration **(B)** following 2% GBT (white columns), 0.4% BNX (black columns) or GBT after BNX pretreatment (gray columns). Data are plotted as mean ± SEM. The dotted line indicates the basal value. Differences between groups were tested for statistical significance using two-way ANOVA with Bonferroni multiple comparison post-test. *p < 0.05 relative to basal (*n* = 6 eyes).

To investigate further this unexpected effect of GBT eye drops, a more stringent kinetics of aqueous tear secretion and protein concentration was carried out, in order to better evaluate the effects of 2% GBT at different times after instillation, in comparison to a topical anesthetic (0.4% BNX). GBT produced a significant increase in aqueous tear secretion at 15 min, an effect which was maintained up to 90 min (white columns in Figure 3A). This secretagogue activity of GBT resulted in a significant decrease in protein concentration at 15 min, but basal protein levels were restored within 30 min (white columns in Figure 3B). In line with previous findings (Shiono, 1989), BNX reduced aqueous secretion by about 50% at 15 min, but basal levels were also recovered within 30 min (black columns in Figure 3A). The sudden decrease in the aqueous secretion was accompanied by an increase in protein concentration, which also recovered its basal level within 30 min (black columns in Figure 3B). Aqueous tear secretion and protein concentration were also measured following the administration of BNX 15 min before the instillation of GBT. In this protocol, pretreatment with BNX was found to prevent the effect of GBT on both aqueous tear secretion and protein concentration (gray columns in Figures 3A,B).

**FIGURE 4:**
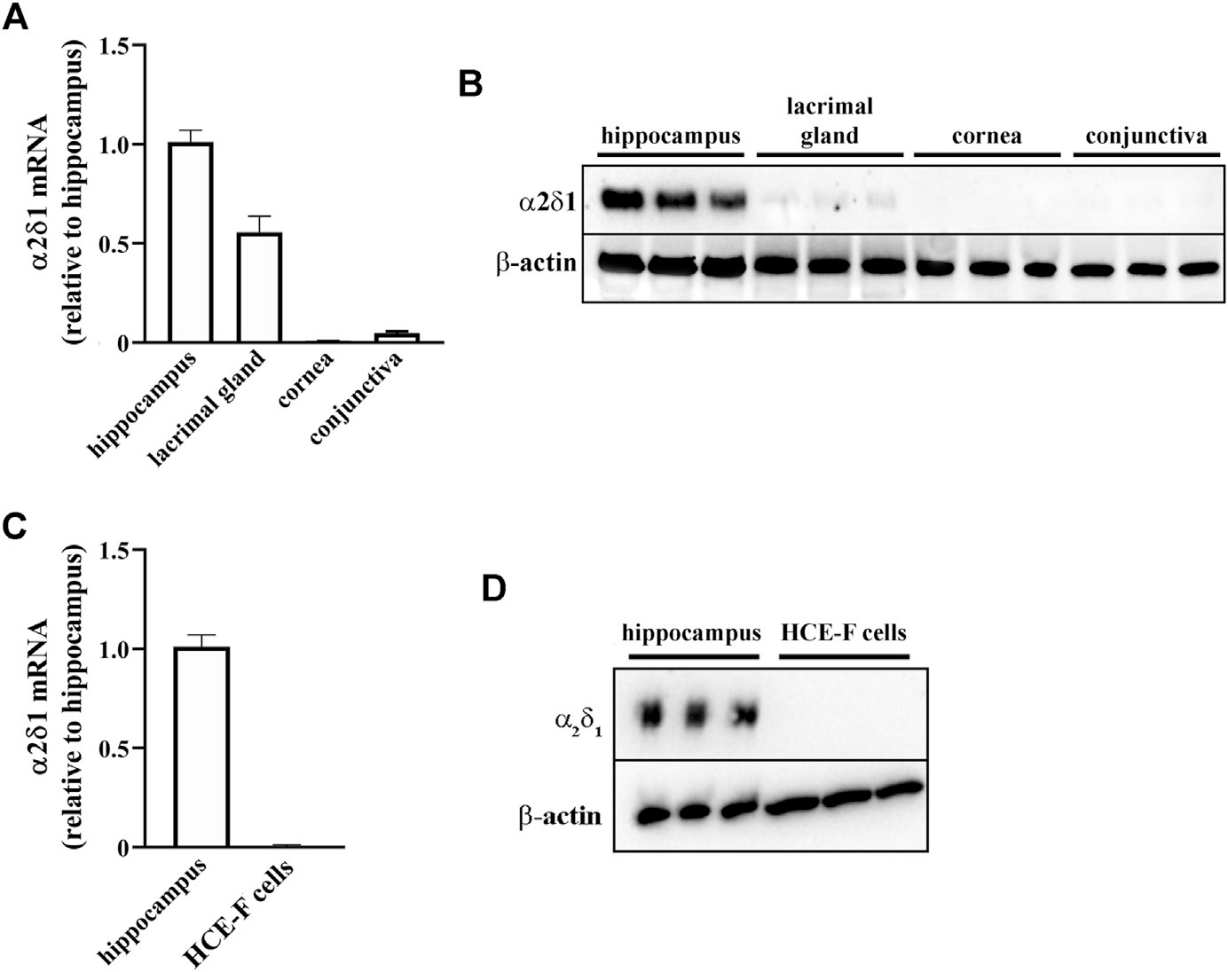
Levels of α2δ1 in rabbit hippocampus, ocular tissues and HCE-F cells. **(A)** Transcript levels of α2δ1 in hippocampus, lacrimal gland, cornea and conjunctiva as determined by qPCR. **(B)** Representative blots depicting levels of α2δ1 in hippocampus, lacrimal gland, cornea and conjunctiva as determined by Western blotting. **(C)** Transcript levels of α2δ1 as determined by qPCR. **(D)** Representative blots depicting levels of α2δ1 in HCE-F as determined by Western blotting. Data are plotted as mean ± SEM (*n* = 6 hippocampus or tissue samples or *n* = 5 HCE-F cells).

To evaluate whether GBT may exert its secretagogue effect through the α2δ1 subunit of the voltage-gated calcium channels, which is a known target of GBT, we evaluated the expression of α2δ1 mRNA and protein in ocular tissues (lacrimal gland, cornea, conjunctiva). As positive control, α2δ1 expression was also evaluated in the hippocampus that is known to express this subunit (Schlick et al., 2010). As shown in Figure 4, α2δ1 was largely expressed in the hippocampus at both mRNA (Figure 4A) and protein (Figure 4B) levels, whereas α2δ1 mRNA was barely detectable in the cornea and conjunctiva (Figure 4A), with no evident protein expression (Figure 4B). In the lacrimal gland α2δ1 mRNA levels were about 50% lower than in the hippocampus (Figure 4A), although a much lower protein expression could be detected (Figure 4B). The evaluation of mRNA and protein expression of α2δ1 in HCE-F cells demonstrated that α2δ1 is not expressed by human primary corneal epithelial cells (Figures 4C,D).

### Gabapentin Effect on Acetylcholine and Norepinephrine Levels in the Lacrimal Gland

**FIGURE 5:**
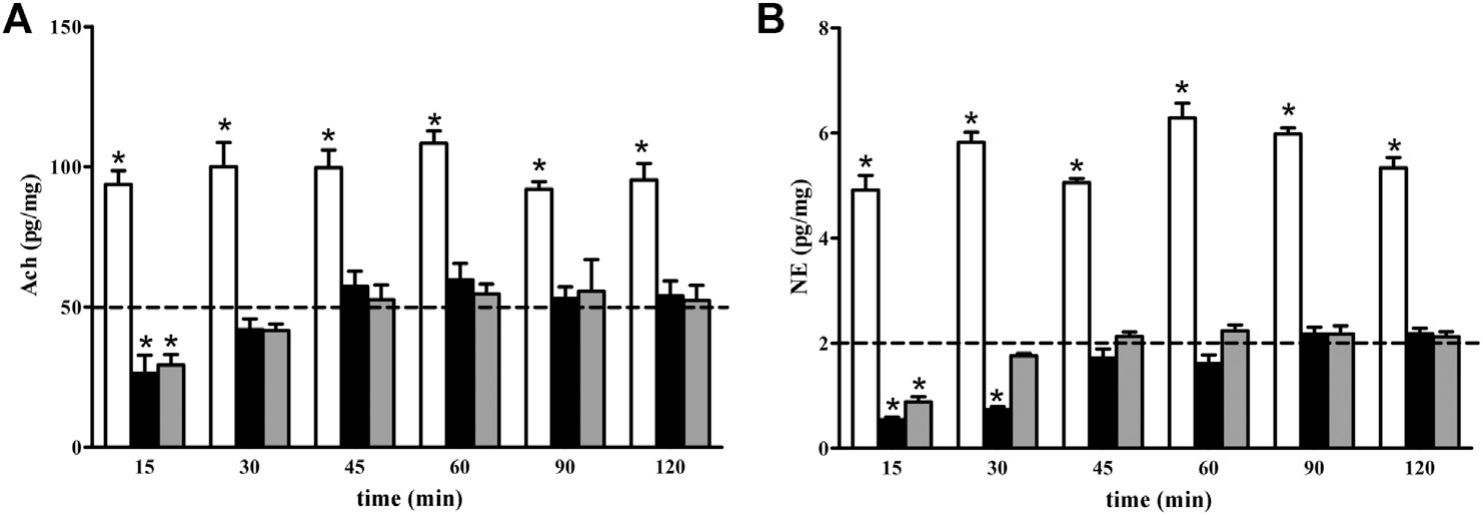
Longitudinal evaluation of Ach **(A)** and NE **(B)** levels following 2% GBT (white columns), 0.4% BNX (black columns) or GBT after BNX pretreatment (gray columns). Data are plotted as mean ± SEM. The dotted line indicates the basal value. Differences between groups were tested for statistical significance using two-ways ANOVA with Bonferroni multiple comparison post-test. *p < 0.05 relative to basal, (*n* = 6 lacrimal glands).

As shown in Figure 5, the levels of both Ach (Figure 5A) and NE (Figure 5B) were increased 15 min after GBT, an effect that persisted until 120 min (white columns in Figures 5A,B). On the contrary, BNX reduced Ach and NE levels with an effect that persisted up to 15 and 30 min, respectively, (black columns in Figures 5A,B). BNX pretreatment 15 min before GBT instillation prevented the GBT-induced increase in Ach and NE over the first 15 min after which basal levels were recovered (gray columns in Figures 5A,B).

### Gabapentin Regulation of Aquaporin 5 Levels in the Lacrimal Gland

**FIGURE 6:**
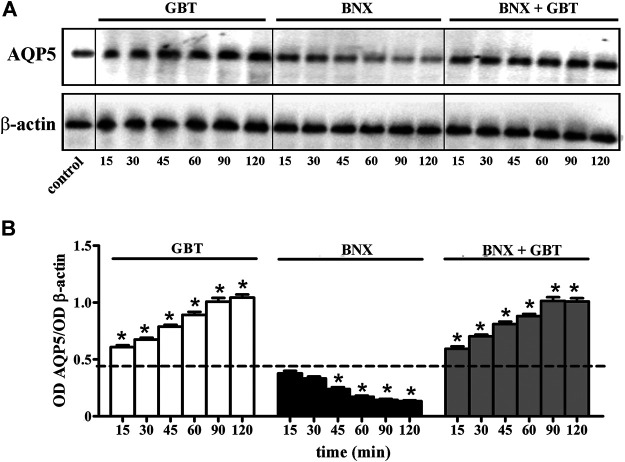
Longitudinal evaluation of AQP5 levels in the lacrimal gland following 2% GBT, 0.4% BNX or GBT after BNX pretreatment. **(A)** Representative blots depicting levels of AQP5 as determined by Western blotting. **(B)** Densitometric analysis of AQP5 levels. The expression of AQP5 was relative to the loading control β-actin. Data are plotted as mean ± SEM. The dotted line indicates the basal value. Differences between groups were tested for statistical significance using one-way ANOVA with Tukey’s multiple comparison post-test. *p < 0.05 relative to basal (*n* = 6 lacrimal glands).

We evaluated whether the increased aqueous tear secretion induced by GBT might involve altered expression of AQP5 in the lacrimal gland. As shown in Figure 6, GBT progressively increased AQP5 levels to reach about a 2-fold increase within 120 min after instillation. BNX instillation resulted in decreased AQP5 levels that at 120 min were still about 70% lower than the basal level. BNX pretreatment 15 min before GBT did not prevent the GBT-induced increase in AQP5 levels.

### Gabapentin Regulation of Aquaporin 5 Levels in Human Corneal Epithelial Cells

**FIGURE 7:**
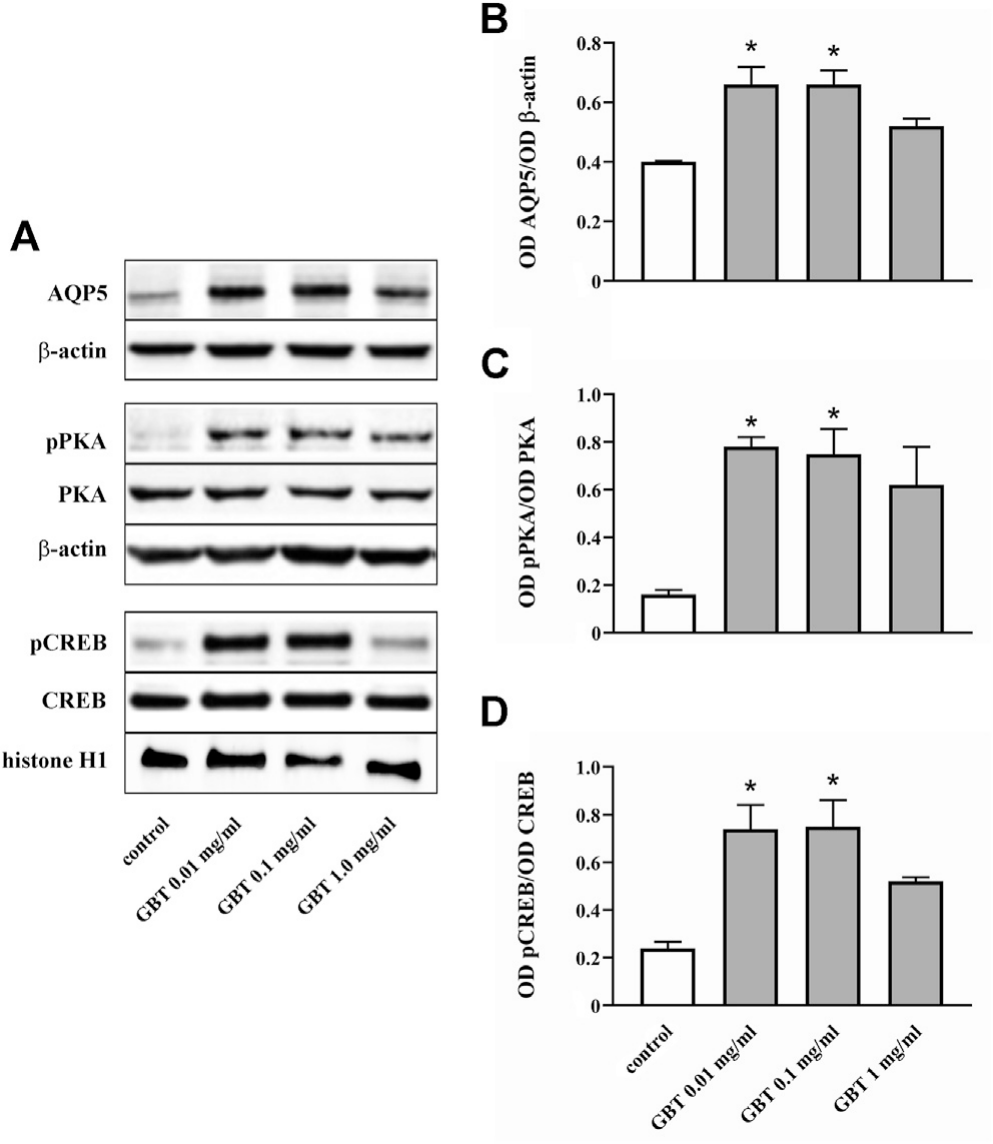
Level of AQP5 and downstream mediators in HCE-F cells treated with GBT at 0.01, 0.1 or 1 mg/ml. **(A)** Representative blots depicting levels of AQP5, pPKA, PKA, pCREB, and CREB after treatment with GBT as determined by Western blotting. **(B-D)** Densitometric analysis of the respective levels. The expression of AQP5 was relative to the loading control β-actin, while the expression of pPKA and pCREB was normalized to the level of PKA and CREB, respectively. Data are plotted as mean ± SEM. Differences between groups were tested for statistical significance using one-way ANOVA with Tukey’s multiple comparison post-test. *p < 0.05 relative to control (*n* = 5).

**FIGURE 8:**
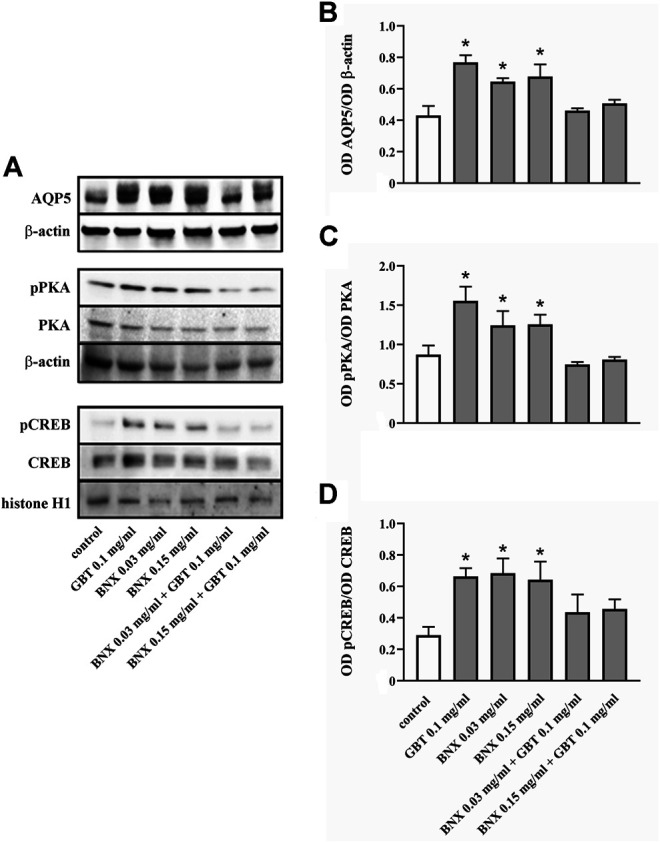
Level of AQP5 and downstream mediators in HCE-F cells treated with 0.1 mg/ml GBT or BNX at 0.03 or 0.15 mg/ml, either alone or in combination. **(A)** Representative blots depicting levels of AQP5, pPKA, PKA, pCREB, and CREB after treatment with GBT, BNX either alone or in combination as determined by Western blotting. **(B–D)** Densitometric analysis of the respective levels. The expression of AQP5 was relative to the loading control β-actin, while the expression of pPKA and pCREB was normalized to the level of PKA and CREB, respectively. Data are plotted as mean ± SEM. Differences between groups were tested for statistical significance using one-way ANOVA with Tukey’s multiple comparison post-test. *p < 0.05 relative to untreated (*n* = 5).

Whether GBT affects AQP5 expression at the corneal level was evaluated by an *in vitro* model of primary human corneal epithelial cells, the HCE-F model (Cristaldi et al., 2020). As shown in Figures 7A,B GBT treatment for 24 h at 0.01 and 0.1 mg/ml significantly increased AQP5 levels by about 55%, while no significant effects were observed after GBT at 1.0 mg/ml. AQP5 expression in epithelial cells is known to involve the PKA/CREB pathway that positively regulates both expression and localization of AQP5 (Yang et al., 2003; Kumari et al., 2012). Whether GBT-induced upregulation of AQP5 might be coupled to the PKA/CREB pathway was then investigated. As shown in Figures 7A,C,D, GBT at 0.01 and 0.1 mg/ml increased PKA phosphorylation by about 5-fold, while the phosphorylated form of the nuclear CREB was increased by about 3-fold. GBT at 1.0 mg/ml had no effect. In Figure 8, the respective effects of GBT and BNX on AQP5 expression were addressed. BNX administration at 0.03 and 0.15 mg/ml resulted in increased levels of AQP5 – similar to GBT–without significant differences between the two concentrations. Consistent with the increase of AQP5, BNX also elevated the amounts of pPKA and pCREB, to levels similar to those obtained with GBT. The association of GBT (0.1 mg/ml) and BNX (0.03 or 0.15 mg/ml) did not influence the expression of neither AQP5 nor the signaling molecules pPKA and pCREB.

## Discussion

We have shown that topical GBT attenuates ocular pain in a rabbit model of corneal injury induced by formaldehyde administration to the cornea through an analgesic but not anesthetic mechanism. However–differently from a topical anesthetic drug such as BNX, which reduces lacrimation–GBT rather stimulates tear secretion by exerting a regulatory role on autonomic neurotransmission and AQP5 expression levels in the lacrimal gland, through a mechanism that seems to be independent from its main receptor α2δ1. Results from *in vitro* experiments using HCE-F cells, a model of human corneal epithelial cells, show that GBT treatment induces AQP5 overexpression by involving the PKA/CREB pathway. This suggests the possibility that, at least at the corneal level, GBT may influence tear secretion by a direct effect on AQP5 expression.

### Analgesic and Secretagogue Effects of Gabapentin

GBT systemic administration has therapeutic efficacy for neurological and psychiatric disorders such as epilepsy, anxiety, and migraine, and, together with its derivatives, belongs to the most used drugs for neuropathic pain management (Fornasari, 2017). The main target of GBT is the α2δ1 auxiliary subunit of presynaptic voltage-gated calcium channels through which GBT reduces the release of multiple excitatory neurotransmitters thus decreasing neuropathic pain (Taylor and Harris, 2020). In addition, there is evidence that GBT may play a role in reducing nociceptive pain (Hamidi et al., 2014; Scuteri et al., 2020). However, the exact mechanism through which GBT, by involving multiple players, exerts its analgesic effect is complex and not definitively clarified (Taylor and Harris, 2020). In ocular neuropathies, systemic administration of GBT or its analog pregabalin has been demonstrated to reduce pain (Lichtinger et al., 2011; Pakravan et al., 2012; Wei et al., 2015; Ongun and Ongun, 2019; Yoon et al., 2020), although its efficacy in counteracting ocular pain in patients with DED has been questioned (Ozmen, 2020).

The present finding that protein levels of the α2δ1 subunit of voltage-gated calcium channels are absent in ocular tissues is not unexpected, taking into consideration the effects of GBT observed here. It is indeed known that the α2δ1-mediated effects of GBT result in reduced neurotransmitter release (Taylor and Harris, 2020), while our results demonstrate increased levels of both Ach and NE in the lacrimal gland. This finding suggests that targets different from the α2δ1 subunit are involved in mediating the effects of GBT (on both pain and tear secretion) when topically applied. The additional finding that in the lacrimal gland protein levels of the α2δ1 subunit are almost absent despite the presence of α2δ1 transcripts is in line with similar studies (Gao et al., 2001; Gong et al., 2001; Dolphin, 2013), suggesting that the expression of α2δ genes may be subjected to post-transcriptional regulation. The possibility remains that GBT may act to relieve pain on the terminal endings that densely innervate the corneal surface (Galor et al., 2015) and are likely to be damaged by formaldehyde used here to induce ocular sensibilization. This possibility will be investigated in future work. An attractive hypothesis is that the stimulation of tear secretion by GBT may explain at least in part its efficacy in counteracting ocular pain, as an increase in tear secretion may improve the lubricating effect of tears on the ocular surface. In fact, among the options available to treat patients suffering from DED, tear replacement is widely used to restore the original homeostasis of the ocular surface and to attenuate patient discomfort and pain, for instance by reducing the friction on the ocular surface, as attrition consequent to lubrication deficits has been recognized to impact on ocular pain (van Setten, 2020). In this respect, artificial tears based on hyaluronic acid have been used since the early 1990 to alleviate dry eye signs and symptoms in DED patients thanks to its water retention and lubricant properties (Yang et al., 2021). On the other hand, there is evidence that artificial tears that solely swell and absorb water show poor effects when applied to DED patients affected by neuropathic pain (Galor et al., 2016), suggesting that those patients without neuropathic pain are more likely to benefit from tear replacement. As shown for the first time by the present results, the analgesic effect of GBT is coupled to a secretagogue activity, an effect which is prevented by the administration of the anesthetic BNX, at least soon after its administration. In this respect, more work should be done to clarify the mechanisms behind the results of their interaction, in particular because BNX and GBT express a completely different mode of action, with BNX able to block sodium channels and to prevent synaptic transmission, supporting the involvement of different downstream mechanisms modulated by BNX and GBT.

As also shown by the present findings, at 15 min after GBT instillation there is an increase in tear secretion paralleled by a decrease in tear protein concentration. This may be explained by assuming that the increase in water secretion follows a faster kinetics than the increase in protein secretion. Therefore, at 15 min the GBT-dependent increase in water secretion dilutes tears, an effect that is no more detectable at increasing time due to the GBT-dependent increase in protein secretion that would parallel water secretion thus restoring a normal tear protein concentration.

### Mechanisms Underlying Gabapentin-Associated Tear Secretion

The autonomic nervous system acts on both the cornea and the lacrimal gland by regulating tear secretion through the release of its main neurotransmitters of which Ach mostly influences water secretion, while NE regulates protein secretion (Dartt, 2009). As shown by the present results, GBT causes upregulation of both Ach and NE levels in the lacrimal gland suggesting that GBT may act on lacrimation through a modulation of the autonomic neurotransmission. On the other hand, since a crude homogenate of lacrimal glands has been used here, we cannot be sure that NE and Ach are actually released by the autonomic nervous system as their origin may be diverse. However, it is unlikely that they might come, for instance, from the plasma permeating the tissue, in which NE levels in the rabbit are about 1 ng/ml (Yokoyama et al., 1992), because, if their origin is systemic from the blood stream, this would hardly explain the local sudden increase (within 15 min) of both neuromodulators elicited by GBT. If confirmed by further experiments, this would be the first demonstration of GBT effects on the autonomic innervation at the level of the eye, while some interactions between GBT and the autonomic nervous system had already been shown (Tanabe et al., 2005; Takasu et al., 2006; Hayashida et al., 2007; Hayashida et al., 2008).

The possibility that GBT may exert a secretagogue role is presently unknown. The only mention about a link between GBT and secretagogue activity (however of the inhibition type) can be found in a case report of a patient with aquagenic wrinkling of the palms describing ameliorative effects of GBT treatment presumably through GBT action on sodium retention by epidermal cells thus promoting skin drying (Emiroglu et al., 2017).

Considering our model, the simplest explanation of the secretagogue action of GBT is that autonomic modulation of tear secretion involves water channels among which aquaporins are likely to play an important role. Some information about enhanced aqueous tear secretion and aquaporins dates back to 1997 when increased levels of AQP5 have been determined in the lacrimal glands of mice after parasympathetic stimulation by pilocarpine (Ishida et al., 1997). In the lacrimal gland, AQP5 is mainly localized at acinar cells, although abundant AQP5 also appears to be localized in the lacrimal duct system (Ding et al., 2010). At the functional level, reduced expression of AQP5 has been determined in the lacrimal gland of patients with Sjögren syndrome, an autoimmune pathology characterized by extreme eye dryness (Tsubota et al., 2001). In addition, abnormal levels of AQP5 are present in tears of patients with dry eye indicating that AQP5 leaks into the lacrimal fluid from damaged cells of the lacrimal gland or the cornea (Ohashi et al., 2003). In animal models, reduced levels of AQP5 have been measured in the lacrimal glands of pregnant rabbits characterized by DED (Ding et al., 2011).

Little is known on AQP5 regulation by autonomic innervation in the lacrimal gland. Recently, in models of DED, PACAP has been reported to induce tear secretion by promoting a PKA-mediated upregulation of AQP5 (Nakamachi et al., 2016). Our results suggest that AQP5 expression is a necessary, however not sufficient condition for increased tear secretion. In fact, GBT increases both lacrimation and AQP5 expression, whereas the effects of BNX on lacrimation are uncoupled from AQP5 expression, since the decrease in lacrimation happens and is concluded before AQP5 decrease. Moreover, when BNX is given soon before GBT, the secretagogue effect of GBT disappears despite the progressive increase of AQP5, supporting the involvement of different targets for BNX and GBT.

In addition to the lacrimal gland, AQP5 is also expressed in the corneal epithelium (Raina et al., 1995) where it is responsible for water movement to the ocular surface (Verkman et al., 2008). In particular, deletion of AQP5 reduces corneal water permeability (Thiagarajah and Verkman, 2002) thus causing marked tear film hypertonicity due to its role as a major component of an osmotically-driven water pathway that contributes to maintain tear isotonicity (Thiagarajah and Verkman, 2002; Ruiz-Ederra et al., 2009).

Results obtained by using HCE-F cells as an *in vitro* model of corneal epithelial cells show that GBT upregulates AQP5 expression via a signaling pathway that involves the activation of the PKA/CREB pathway, thus suggesting that GBT may affect tear secretion also by acting at the corneal level. The stimulatory effect of GBT on AQP5 expression and its pathway of activation tends to decrease with the increasing amount of GBT, and peaks at the two lower doses of 0.01 and 0.1 mg/ml, in line with what expected for a ligand showing a hormetic-like biphasic dose response in a ligand-receptor interaction (Calabrese, 2013). Surprisingly, and in contrast to what observed in the lacrimal gland, also BNX stimulates AQP5 expression in corneal epithelial cells through the same pathway activated by GBT (PKA/CREB), and with the same modality (higher dose, lesser effect), suggesting that *in vivo* there is an interplay between the autonomic nervous system and the regulation of lacrimation and AQP5 expression, while in a cell monolayer *in vitro* the effect is mediated only by biochemical interactions, which apparently use the same pathway to regulate AQP5 expression. Moreover, the association of GBT and BNX *in vitro* results in no stimulation on AQP5 expression and pathway activation. Most likely, given the fact that both compounds impinge on the same regulatory pathway (PKA/CREB), the association of the two might result in an overstimulation of the system, like in the case of high doses of GBT or BNX, finally resulting in a lesser response in terms of activation.

In conclusion, the present data demonstrate the analgesic and not anesthetic effect of a topical formulation of GBT as eye drops, and for the first time show a secretagogue effect of GBT, that is likely to involve both a stimulation of the autonomic nervous system and a direct activation of intracellular signaling cascades, including the PKA/CREB pathway, culminating in the increased expression of AQP5. However, whether this effect may also involve a modulation of ion channels remains to be determined as no evaluation of transmembrane ion movement/potential has been performed in the present study. Therefore, future mechanistic investigations will be therefore required to decipher the targets through which GBT acts to increase tear secretion. Overall, the presence in the same molecule of analgesic, anti-inflammatory (Anfuso et al., 2017) and secretagogue effects suggest a useful application of GBT eye drops in the treatment of medium/severe dry eye, in which the algic and inflammatory components accompany tear deficiency.

## Data Availability

The original contributions presented in the study are included in the article/Supplementary Material, further inquiries can be directed to the corresponding author.

## References

[B1] Anfuso C. D. Olivieri M. Fidilio A. Lupo G. Rusciano D. Pezzino S. (2017). Gabapentin Attenuates Ocular Inflammation: *In Vitro* and *In Vivo* Studies. Front. Pharmacol. 8, 173. 10.3389/fphar.2017.00173 28420991 PMC5378778

[B2] Calabrese E. J. (2013). Biphasic Dose Responses in Biology, Toxicology and Medicine: Accounting for Their Generalizability and Quantitative Features. Environ. Pollut. 182, 452–460. 10.1016/j.envpol.2013.07.046 23992683

[B3] Cristaldi M. Olivieri M. Spampinato G. Anfuso C. D. Scalia M. Lupo G. (2020). Isolation and Characterization of a New Human Corneal Epithelial Cell Line: HCE-F. Cornea 39, 1419–1425. 10.1097/ICO.0000000000002357 32452988

[B4] Dartt D. A. (2009). Neural Regulation of Lacrimal Gland Secretory Processes: Relevance in Dry Eye Diseases. Prog. Retin. Eye Res. 28, 155–177. 10.1016/j.preteyeres.2009.04.003 19376264 PMC3652637

[B5] Delporte C. (2009). Aquaporins in Secretory Glands and Their Role in Sjögren's Syndrome. Handb. Exp. Pharmacol. 190, 185–201. 10.1007/978-3-540-79885-9_9 19096778

[B6] Ding C. Lu M. Huang J. (2011). Changes of the Ocular Surface and Aquaporins in the Lacrimal Glands of Rabbits during Pregnancy. Mol. Vis. 17, 2847–2855 . 22128232 PMC3224838

[B7] Ding C. Parsa L. Nandoskar P. Zhao P. Wu K. Wang Y. (2010). Duct System of the Rabbit Lacrimal Gland: Structural Characteristics and Role in Lacrimal Secretion. Invest. Ophthalmol. Vis. Sci. 51, 2960–2967. 10.1167/iovs.09-4687 20107177 PMC2891459

[B8] Dolphin A. C. (2013). The α2δ Subunits of Voltage-Gated Calcium Channels. Biochim. Biophys. Acta (Bba) - Biomembranes 1828, 1541–1549. 10.1016/j.bbamem.2012.11.019 23196350

[B9] Emiroglu N. Cengiz F. P. Su O. Onsun N. (2017). Gabapentin-induced Aquagenic Wrinkling of the Palms. Dermatol. Online J. 23, 13030. 28329484

[B10] Fornasari D. (2017). Pharmacotherapy for Neuropathic Pain: A Review. Pain Ther. 6 (Suppl. 1), 25–33. 10.1007/s40122-017-0091-4 29178034 PMC5701897

[B11] Galor A. Batawi H. Felix E. R. Margolis T. P. Sarantopoulos K. D. Martin E. R. (2016). Incomplete Response to Artificial Tears Is Associated with Features of Neuropathic Ocular Pain. Br. J. Ophthalmol. 100, 745–749. 10.1136/bjophthalmol-2015-307094 26377416

[B12] Galor A. Levitt R. C. Felix E. R. Martin E. R. Sarantopoulos C. D. (2015). Neuropathic Ocular Pain: an Important yet Underevaluated Feature of Dry Eye. Eye 29, 301–312. 10.1038/eye.2014.263 25376119 PMC4366454

[B13] Galor A. (2019). Painful Dry Eye Symptoms: A Nerve Problem or a Tear Problem?. Ophthalmology 126, 648–651. 10.1016/j.ophtha.2019.01.028 31005185

[B14] Gao T. Cuadra A. E. Ma H. Bünemann M. Gerhardstein B. L. Cheng T. (2001). C-terminal Fragments of the α1C(CaV1.2) Subunit Associate with and Regulate L-type Calcium Channels Containing C-Terminal-Truncated α1CSubunits. J. Biol. Chem. 276, 21089–21097. 10.1074/jbc.M008000200 11274161

[B15] Gong H. C. Hang J. Kohler W. Li L. Su T.-Z. (2001). Tissue-specific Expression and Gabapentin-Binding Properties of Calcium Channel α2δ Subunit Subtypes. J. Membr. Biol. 184, 35–43. 10.1007/s00232-001-0072-7 11687876

[B16] Hamidi G. A. Jafari-Sabet M. Abed A. Mesdaghinia A. Mahlooji M. Banafshe H. R. (2014). Gabapentin Enhances Anti-nociceptive Effects of Morphine on Heat, Cold, and Mechanical Hyperalgesia in a Rat Model of Neuropathic Pain. Iran J. Basic Med. Scij. Basic Med. Sci. 17, 753–759. PMC4340982 25729543

[B17] Hayashida K.-i. DeGoes S. Curry R. Eisenach J. C. (2007). Gabapentin Activates Spinal Noradrenergic Activity in Rats and Humans and Reduces Hypersensitivity after Surgery. Anesthesiology 106, 557–562. 10.1097/00000542-200703000-00021 17325515

[B18] Hayashida K.-i. Obata H. Nakajima K. Eisenach J. C. (2008). Gabapentin Acts within the Locus Coeruleus to Alleviate Neuropathic Pain. Anesthesiology 109, 1077–1084. 10.1097/ALN.0b013e31818dac9c 19034104 PMC2843419

[B19] Honkanen R. Nemesure B. Huang L. Rigas B. (2021). Diagnosis of Dry Eye Disease Using Principal Component Analysis: A Study in Animal Models of the Disease. Curr. Eye Res. 46, 622–629. 10.1080/02713683.2020.1830115 33445973

[B20] Ishida N. Hirai S.-I. Mita S. (1997). Immunolocalization of Aquaporin Homologs in Mouse Lacrimal Glands. Biochem. Biophysical Res. Commun. 238, 891–895. 10.1006/bbrc.1997.7396 9325187

[B21] Jacobs D. S. (2017). Diagnosis and Treatment of Ocular Pain: the Ophthalmologist's Perspective. Curr. Ophthalmol. Rep. 5, 271–275. 10.1007/s40135-017-0152-1 29226029 PMC5711963

[B22] Joubert F. Acosta M. d. C. Gallar J. Fakih D. Sahel J.-A. Baudouin C. (2019). Effects of Corneal Injury on Ciliary Nerve Fibre Activity and Corneal Nociception in Mice: A Behavioural and Electrophysiological Study. Eur. J. Pain 23, 589–602. 10.1002/ejp.1332 30370980

[B23] Kavalieratos C.-S. Dimou T. (2008). Gabapentin Therapy for Painful, Blind Glaucomatous Eye: Case Report. Pain Med. 9, 377–378. 10.1111/j.1526-4637.2006.00167.x 18366517

[B24] Kumari S. S. Varadaraj M. Yerramilli V. S. Menon A. G. Varadaraj K. (2012). Spatial Expression of Aquaporin 5 in Mammalian Cornea and Lens, and Regulation of its Localization by Phosphokinase A. Mol. Vis. 18, 957–967. 22550388 PMC3340213

[B25] Lai L.-J. Hsu W.-H. Wu A. M. Wu J. H. (2013). Ocular Injury by Transient Formaldehyde Exposure in a Rabbit Eye Model. PLoS One 8, e66649. 10.1371/journal.pone.0066649 23818956 PMC3688594

[B26] Lichtinger A. Purcell T. L. Schanzlin D. J. Chayet A. S. (2011). Gabapentin for Postoperative Pain after Photorefractive Keratectomy: a Prospective, Randomized, Double-Blind, Placebo-Controlled Trial. J. Refract. Surg. 27, 613–617. 10.3928/1081597X-20110210-01 21366172

[B27] Meng I. D. Kurose M. (2013). The Role of Corneal Afferent Neurons in Regulating Tears under normal and Dry Eye Conditions. Exp. Eye Res. 117, 79–87. 10.1016/j.exer.2013.08.011 23994439 PMC3989072

[B28] Nakamachi T. Ohtaki H. Seki T. Yofu S. Kagami N. Hashimoto H. (2016). PACAP Suppresses Dry Eye Signs by Stimulating Tear Secretion. Nat. Commun. 7, 12034. 10.1038/ncomms12034 27345595 PMC4931240

[B29] Ohashi Y. Ishida R. Kojima T. Goto E. Matsumoto Y. Watanabe K. (2003). Abnormal Protein Profiles in Tears with Dry Eye Syndrome. Am. J. Ophthalmol. 136, 291–299. 10.1016/s00029394(03)00203-410.1016/s0002-9394(03)00203-4 12888052

[B30] Ongun N. Ongun G. T. (2019). Is Gabapentin Effective in Dry Eye Disease and Neuropathic Ocular Pain?. Acta Neurol. Belg. 121, 397–401. 10.1007/s13760-019-01156-w 31134508

[B31] Ozmen M. C. (2020). Is Gabapentin Effective in Dry Eye Disease and Neuropathic Ocular Pain?. Acta Neurol. Belg. 120, 1215–1216. 10.1007/s13760-019-01251-y 31776811

[B32] Pakravan M. Roshani M. Yazdani S. Faramazi A. Yaseri M. (2012). Pregabalin and Gabapentin for post-photorefractive Keratectomy Pain: a Randomized Controlled Trial. Eur. J. Ophthalmol. 22 (Suppl. 7), 106–113. 10.5301/ejo.5000143 22577038

[B33] Raina S. Preston G. M. Guggino W. B. Agre P. (1995). Molecular Cloning and Characterization of an Aquaporin cDNA from Salivary, Lacrimal, and Respiratory Tissues. J. Biol. Chem. 270, 1908–1912. 10.1074/jbc.270.4.1908 7530250

[B34] Ruiz-Ederra J. Levin M. H. Verkman A. S. (2009). *In Situ* Fluorescence Measurement of Tear Film [Na+], [K+], [Cl−], and pH in Mice Shows Marked Hypertonicity in Aquaporin-5 Deficiency. Invest. Ophthalmol. Vis. Sci. 50, 2132–2138. 10.1167/iovs.08-3033 19136711 PMC2904304

[B35] Russo A. F. (2017). Overview of Neuropeptides: Awakening the Senses?. Headache: J. Head Face Pain 57 (Suppl. 2), 37–46. 10.1111/head.13084 PMC5424629 28485842

[B36] Santagati M. G. La Terra Mulè S. Amico C. Pistone M. Rusciano D. Enea V. (2005). Lactoferrin Expression by Bovine Ocular Surface Epithelia: a Primary Cell Culture Model to Study Lactoferrin Gene Promoter Activity. Ophthalmic Res. 37, 270–278. 10.1159/000087372 16103737

[B37] Schlick B. Flucher B. E. Obermair G. J. (2010). Voltage-activated Calcium Channel Expression Profiles in Mouse Brain and Cultured Hippocampal Neurons. Neuroscience 167, 786–798. 10.1016/j.neuroscience.2010.02.037 20188150 PMC3315124

[B38] Scuteri D. Berliocchi L. Rombolà L. Morrone L. A. Tonin P. Bagetta G. (2020). Effects of Aging on Formalin-Induced Pain Behavior and Analgesic Activity of Gabapentin in C57BL/6 Mice. Front. Pharmacol. 11, 663. 10.3389/fphar.2020.00663 32457634 PMC7227482

[B39] Shiono T. (1989). Effect of Topical Anesthesia on Secretion of Lysozyme and Lysosomal Enzymes in Human Tears. Jpn. J. Ophthalmol. 33, 375–379. 2796016

[B40] Struble C. Howard S. Relph J. (2014). Comparison of Ocular Tissue Weights (Volumes) and Tissue Collection Techniques in Commonly Used Preclinical Animal Species. Acta Ophthalmol. 92, S005. 10.1111/j.1755-3768.2014.S005.x

[B41] Takasu K. Honda M. Ono H. Tanabe M. (2006). Spinal α 2 -adrenergic and Muscarinic Receptors and the NO Release cascade Mediate Supraspinally Produced Effectiveness of Gabapentin at Decreasing Mechanical Hypersensitivity in Mice after Partial Nerve Injury. Br. J. Pharmacol. 148, 233–244. 10.1038/sj.bjp.0706731 16582934 PMC1617063

[B42] Tanabe M. Takasu K. Kasuya N. Shimizu S. Honda M. Ono H. (2005). Role of Descending Noradrenergic System and Spinal α 2 -adrenergic Receptors in the Effects of Gabapentin on thermal and Mechanical Nociception after Partial Nerve Injury in the Mouse. Br. J. Pharmacol. 144, 703–714. 10.1038/sj.bjp.0706109 15678083 PMC1576051

[B43] Taylor C. P. Harris E. W. (2020). Analgesia with Gabapentin and Pregabalin May Involve N-Methyl-D-Aspartate Receptors, Neurexins, and Thrombospondins. J. Pharmacol. Exp. Ther. 374, 161–174. 10.1124/jpet.120.266056 32321743

[B44] Thiagarajah J. R. Verkman A. S. (2002). Aquaporin Deletion in Mice Reduces Corneal Water Permeability and Delays Restoration of Transparency after Swelling. J. Biol. Chem. 277, 19139–19144. 10.1074/jbc.M202071200 11891232

[B45] Tsubota K. Hirai S. King L. S. Agre P. Ishida N. (2001). Defective Cellular Trafficking of Lacrimal Gland Aquaporin-5 in Sjögren's Syndrome. The Lancet 357, 688–689. 10.1016/S01406736(00)04140-410.1016/s0140-6736(00)04140-4 11247557

[B46] van Setten G.-B. (2020). Impact of Attrition, Intercellular Shear in Dry Eye Disease: When Cells Are Challenged and Neurons Are Triggered. Int. J. Mol. Sci. 21, 4333. 10.3390/ijms21124333 PMC7352662 32570730

[B47] Verkman A. S. (2003). Role of Aquaporin Water Channels in Eye Function. Exp. Eye Res. 76, 137–143. 10.1016/s0014-4835(02)00303-2 12565800

[B48] Verkman A. S. Ruiz-Ederra J. Levin M. H. (2008). Functions of Aquaporins in the Eye. Prog. Retin. Eye Res. 27, 420–433. 10.1016/j.preteyeres.2008.04.001 18501660 PMC3319433

[B49] Wang W. Zheng M. (2011). Role of cAMP-PKA/CREB Pathway in Regulation of AQP 5 Production in Rat Nasal Epithelium. Rhinology 49, 464–469. 10.4193/Rhino10.107 21991573

[B50] Wei L. A. Davies B. W. Hink E. M. Durairaj V. D. (2015). Perioperative Pregabalin for Attenuation of Postoperative Pain after Eyelid Surgery. Ophthalmic Plast. Reconstr. Surg. 31, 132–135. 10.1097/IOP.0000000000000219 25000214

[B51] Yang F. Kawedia J. D. Menon A. G. (2003). Cyclic AMP Regulates Aquaporin 5 Expression at Both Transcriptional and post-transcriptional Levels through a Protein Kinase A Pathway. J. Biol. Chem. 278, 32173–32180. 10.1074/jbc.M305149200 12783871

[B52] Yang Y. J. Lee W. Y. Kim Y. J. Hong Y. P. (2021). A Meta-Analysis of the Efficacy of Hyaluronic Acid Eye Drops for the Treatment of Dry Eye Syndrome. Int. J. Environ. Res. Public Health 18, 2383. 10.3390/ijerph18052383 33804439 PMC7967738

[B53] Yokoyama Y. Uchida M. Matsumoto S. Saito K. Fukuda M. (1992). Changes in Plasma Catecholamine Levels Following Injection of Prostaglandin F2alpha into the Basal Cistern in Rabbits. J. Anesth. 6, 161–166. 10.1007/s0054020060161 15278560

[B54] Yoon H. J. Kim J. Yoon K. C. (2020). Treatment Response to Gabapentin in Neuropathic Ocular Pain Associated with Dry Eye. J. Clin. Med. 9, 3765. 10.3390/jcm9113765 PMC7700262 33266439

[B55] Yu V. Bhattacharya D. Webster A. Bauskar A. Flowers C. Heur M. (2018). Clusterin from Human Clinical Tear Samples: Positive Correlation between Tear Concentration and Schirmer Strip Test Results. Ocul. Surf. 16, 478–486. 10.1016/j.jtos.2018.08.001 30077709 PMC6175631

